# A streamlined workflow for a fast and cost-effective count of tyndallized probiotics using flow cytometry

**DOI:** 10.3389/fmicb.2024.1389069

**Published:** 2024-05-03

**Authors:** Veronica Bolzon, Michela Bulfoni, Massimo Pesando, Alessandro Nencioni, Emanuele Nencioni

**Affiliations:** ^1^Biofarma Group Srl, Udine, Italy; ^2^Department of Medicine, University of Udine, Udine, Italy; ^3^IBSA Institut Biochimique SA, Lugano, Switzerland

**Keywords:** flow cytometry, tyndallized probiotics, quality control, dead microorganisms, bacterial lysates, health benefits

## Abstract

The use of dead probiotics and their cellular metabolites seems to exhibit immunomodulatory and anti-inflammatory properties, providing protection against pathogens. These inanimate microorganisms, often referred to as tyndallized or heat-killed bacteria, are a new class of probiotics employed in clinical practice. Safety concerns regarding the extensive use of live microbial cells have increased interest in inactivated bacteria, as they could eliminate shelf-life problems and reduce the risks of microbial translocation and infection. Culture-dependent methods are not suitable for the quality assessment of these products, and alternative methods are needed for their quantification. To date, bacterial counting chambers and microscopy have been used for tyndallized bacteria enumeration, but no alternative validated methods are now available for commercial release. The aim of the present study is to design a new method for the qualitative and quantitative determination of tyndallized bacterial cells using flow cytometric technology. Using a live/dead viability assay based on two nucleic acid stains, thiazole orange (TO) and propidium iodide (PI), we optimized a workflow to evaluate bacterial viability beyond the reproduction capacity that provides information about the structural properties and metabolic activities of probiotics on FACSVerse without using beads as a reference. The data obtained in this study represent the first analytical application that works effectively both on viable and non-viable cells. The results provided consistent evidence, and different samples were analyzed using the same staining protocol and acquisition settings. No significant discrepancies were highlighted between the declared specification of commercial strain and the analytical data obtained. For the first time, flow cytometry was used for counting tyndallized bacterial cells as a quality control assessment in probiotic production. This aspect becomes important if applied to medical devices where we cannot boast metabolic but only mechanical activities.

## 1 Introduction

Probiotics, according to the World Health Organization (WHO), are considered non-pathogenic live microorganisms that confer a health benefit on the host (Halpern, 1996; Food and Agriculture Organization and World Health Organization Expert Consultation, 2001; Kataria et al., 2009; Adams, 2010; Gupta et al., 2018). Probiotics are now widely used in dietary supplements, food, and infant formula formulations (Halpern, 1996; Kataria et al., 2009; Adams, 2010; Gupta et al., 2018). The main effects of probiotics include balancing and restoring the gut microbiota, protecting against pathogens, modulating the immune system, and maintaining the integrity of the intestinal barrier (Kataria et al., 2009; Adams, 2010; Taverniti and Guglielmetti, 2011; Bermúdez-Brito et al., 2012; Piqué et al., 2019; Michelutti et al., 2020). Probiotics represent the largest category of dietary supplements worldwide. Key functions of probiotics include providing digestive and immune health benefits, such as producing anti-microbial metabolites, synthesizing B-group vitamins, exerting anti-obesity and anti-diabetic effects, lowering cholesterol, and downregulating oxidative stress. They also help to prevent infections or restore the gut microbiome following treatment with antibiotics (Adams, 2010; Piqué et al., 2019; Michelutti et al., 2020). Furthermore, the health benefits of probiotics are species- or genus-specific and are related to their intrinsic characteristics. These positive effects extend to improvements in bowel disorders, lactose intolerance, diarrhea, and allergies, among other positive effects. The mechanisms by which probiotics can exert their action are enhancement of the epithelial barrier, increased adhesion to the intestinal mucosa, gut immunomodulation, neurotransmitter synthesis, and exclusion of pathogens from adhesion sites (Taverniti and Guglielmetti, 2011; Bermúdez-Brito et al., 2012; Piqué et al., 2019). Recently, a new class of non-vital microorganisms, called tyndallized probiotics, has emerged. These new products containing inanimate bacteria are improving consumer acceptance and increasing food industry production. There is evidence that preparations containing dead cells and their metabolites can also exert relevant biological responses, restoring normal intestinal homeostasis (Xiao et al., 2003; Taverniti and Guglielmetti, 2011; Ozen and Dinleyici, 2015; Lee et al., 2016; De Marco et al., 2018; Warda et al., 2018). Therefore, probiotic viability seems to not be an essential condition to exert a beneficial effect on the host. After the inactivation of bacteria, mainly by heat treatment, dead cells can release bacterial components with key immunomodulating effects and antagonizing properties against pathogens (Xiao et al., 2003; Ozen and Dinleyici, 2015; De Marco et al., 2018). The beneficial properties of killed bacteria have been observed in both *in vitro* and clinical trials, demonstrating their benefits in different indications. Importantly, these benefits are achieved without incurring the risks associated with live microorganisms, while also offering pharmaceutical advantages (Xiao et al., 2003; Ozen and Dinleyici, 2015; Lee et al., 2016; Warda et al., 2018; Piqué et al., 2019).

The production of tyndallized bacteria by industries requires quality control assessment throughout all manufacturing paths. This aspect becomes important if applied to medical devices where we cannot boast metabolic but only mechanical activities. Moreover, the emergence of this new generation of probiotics presents significant challenges for enumeration methods, making it necessary to explore alternative techniques to the traditional plate count. For the determination of tyndallization efficacy and for dead-cell enumeration, no methods are available (Auty et al., 2001; Michelutti et al., 2020). Classical methods basically rely on the use of plate count methods, which are still regarded as the benchmark method for the determination of viability (Alvarez-Barrientos et al., 2000; Amor et al., 2002; Assuncao et al., 2007; Ashraf and Shah, 2011; Chiron et al., 2018). Plate counting, considered the gold standard for the enumeration of probiotics, allows us to measure cultivability, not viability.

The classic culture technique showed some drawbacks related to the fact that it could not give deep insights into process-induced changes in cellular integrity or metabolic activities (Díaz et al., 2010; Davis, 2014). Flow cytometric analysis is a promising method for the simultaneous evaluation of multiple cellular parameters, both structural and functional (Hill et al., 2014; Marlicz et al., 2016; Ou et al., 2017; Burta et al., 2018; Jackson et al., 2019). This approach allows an extended description of the bacterial viability state as well as the identification of heterogeneities within the population. In fact, the plate count allows differentiation only between genera. The variability between species and strains has traditionally been considered a fundamental cornerstone in microbiology.

To date, no single methodology can be universally applied to all probiotic organisms, but an integrated approach is needed, as we have already demonstrated in our previous publication (Bolzon et al., 2022). The use of antibody staining by flow cytometry is a non-standardized approach, considering that few commercial antibodies are available. The in-house production of specie-specific antibodies is a long and complex process that is not feasible in every laboratory. Furthermore, for quality control in production, analytical methods should be robust and reproducible. For this reason, molecular approaches based on PCR or qPCR could represent the most suitable workflow to characterize probiotics at the species level.

The objective of this study was to characterize the behavior of tyndallized probiotics and quantify their amount both in the raw material and in the finished product using flow cytometric analysis. The optimized assay consists of a double staining method with TO and PI to assess the effect of inactivation treatments on bacteria. The optimization and application of a flow cytometric analysis in the field of dead probiotic production were described for the first time.

This protocol allowed a precise characterization of the physiological state of bacteria to better manage probiotic production and estimated the real concentration of tyndallized cells supplied to the customer.

## 2 Materials and methods

### 2.1 Sample composition

A commercial strand of tyndallized *Lactobacillus casei* (*HA-108*) was initially used to design and optimize the analytical parameters in terms of morphological and fluorescent gates.

Subsequently, a commercial raw material was selected to study the matrix effect and to define where exactly tyndallized cell took place during the acquisition. The declared potency of the selected raw material was ≥150E+09 cells/g, determined by microscopic count, corresponding to 278.3E+09 cells/g. This quantification was used as the reference gold standard for the new method.

During protocol optimization, consecutive dilutions from 1,0E-04 to 1,0E-09, corresponding to ≥1,5E+09 to ≥1,5E+02, were evaluated by flow cytometry, and the dilution 1,0E-07 (corresponding to approx. 1,0E+04 cells) was chosen as the most suitable concentration for staining and acquisition of tyndallized raw materials.

### 2.2 Tyndallization process

Bacterial cells were anaerobically cultured in a yeast extract-based medium at 37°C overnight and then centrifuged at 12,500 × *g* for 1 h at room temperature using a continuous filter (*Alfa Laval, Lund, Sweden*). Thereafter, the pellets and the supernatant were concentrated five times using a vacuum concentrator (Dong Yang Machine Industry, Seoul, Korea) under reduced pressure at 80°C and then mixed with dry-sterilized cornstarch powder. The cornstarch was not effective in wrinkle reduction. The mix was then frozen at −45°C and lyophilized to obtain ACT 3302 powder. All these steps of tyndallization were performed by the vendor.

### 2.3 Formulations selected for the method verification

Two different tyndallized probiotic raw materials and two different formulations of finished products were analyzed. The first of the two raw materials analyzed consists of tyndallized *Lactobacillus acidophilus* (HA-122), and the other one is a blend of tyndallized *Lactobacillus acidophilus* HA-122, *Lactobacillus casei* HA-108, *Lactobacillus plantarum* HA-119 and *Streptococcus thermophilus* HA-110. One batch of the first and three different batches of the second were analyzed. Finished product samples consist of two different formulations of tyndallized probiotics in a matrix composed of fruit juices, proteins, sugars as oligosaccharides, and vitamins. Three different batches of the first formulation and four different batches of the second one were analyzed. All analyses were performed in triplicate.

### 2.4 Flow cytometry staining protocol

**Figure 1:**
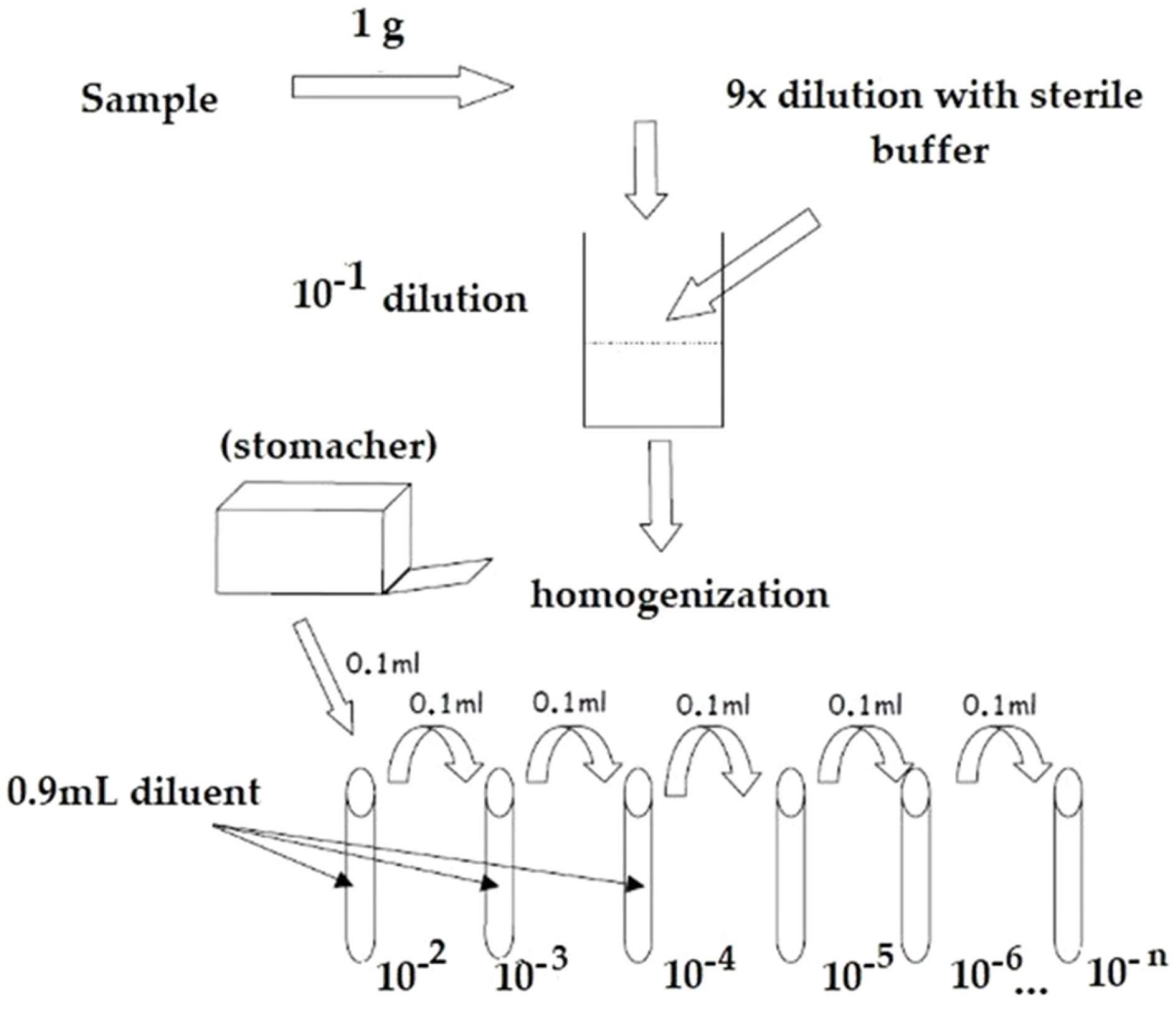
Representative flowchart of the operative workflow employed for the dilution preparation.

A measure of 1 g of probiotic raw material or finished product was initially diluted with 9 mL of appropriate sterile diluent maximum recovery diluent (MRD – *Biolife*), and then consecutive serial decimal dilutions, until appropriate, were performed. The optimal dilutions were used for the flow cytometry staining protocol (see Figure 1).

Two-color staining of probiotic cell suspension using the BD Cell Viability Kit (*BD Biosciences*) was performed. A measure of 500 μL of each dilution was incubated in the dark for 20 min at 37 ± 2°C with 5 μL of 420 nmol/L of thiazole orange (TO), which entered in all cells, and a further 20 min at room temperature with 5 μL of 4.3 μmol/L of propidium iodide (PI), specific for dead cells, following manufacturer’s instructions.

After the incubation, stained samples were analyzed using a FACS Verse (*BD Biosciences*) flow cytometer. A total of 10,000 events were acquired per sample.

#### 2.4.1 Data acquisition and analysis

Initial settings of the flow cytometer were: threshold—FSC 200 arbitrary units (a.u.); logarithmic amplification; FL1, bi-exponential amplification; and FL3, bi-exponential amplification. Automatic compensation was used. Acquired data were analyzed with the FACSuite™ (BD) software in the “Acquisition-to-Analysis” mode. An FSC vs. SSC plot with a physical gate was designed to identify the bacterial population of interest. Then, another specific region in the FL1 (TO) vs. FL3 (PI) plot was gated to display the tyndallized dead stain results. To determine the absolute count, expressed as FU/g, the following equation was used:


N=n×1000×d

(*n* = number of events per μL; *d* = dilution factor corresponding to the dilution).

During the acquisition, the collocation of the events was observed to better define the gates. 1,0E-09 and 1,0E-08 dilutions were excluded because the flow rate of events was too low to clearly separate them from the background; 1,0E-07 and 1,0E-6 proved to be the best ones in terms of count, while dilutions with a large number of events led to underestimated counts, always compared to the microscopic count as the gold standard.

### 2.5 Statistical analysis

The statistic parameters measured were the average, the mean standard deviation (Std Dev), and the confidence interval of 95%. The statistical evaluation of the results has been performed using one-way ANOVA (*p*-value < 0.05). Before applying ANOVA, Levene’s test was performed to verify variances. Statistics were performed by Minitab 19 software.

## 3 Results

### 3.1 Optimization of the analytical parameters

The microscopic count reported in the tyndallized *Lactobacillus casei* HA-108’s certificate of analysis (CoA), corresponding to 278,3E+09 cells/gram, was considered the *gold standard* to refer to all results.

To determine the optimal sample dilution for the flow cytometric analysis, a range from 1,00E-09 to 1,00E-04 tyndallized dilutions was tested.

The flow cytometer settings will vary depending on the cell size being analyzed, and adjustments were necessary. Different threshold settings were evaluated during sample acquisition to observe the best data resolution at different set points and concentrations. The results obtained from serial dilutions of tyndallized *Lactobacillus casei* HA-108, expressed in FU/g, are given in Table 1.

**Table 1:** Results of serial dilutions of *L. casei* obtained during the optimization steps.

Dilution	Replicate 1	Replicate 2	Average
1,00E-09	1,56E+12	4,59E+12	3,08E+12
1,00E-08	5,10E+11	4,58E+11	4,84E+11
**1,00E-07**	**2,71E+11**	**2,46E+11**	**2,59E+11**
1,00E-06	1,66E+11	1,71E+11	1,69E+11
1,00E-05	1,17E+11	1,61E+11	1,39E+11
1,00E-04	9,95E+10	9,93E+10	9,94E+10

Bold values represent the most compliant dilution, according to the microscopic count, that highlighted the cell scatter from background of the matrix effect.

**Figure 2:**
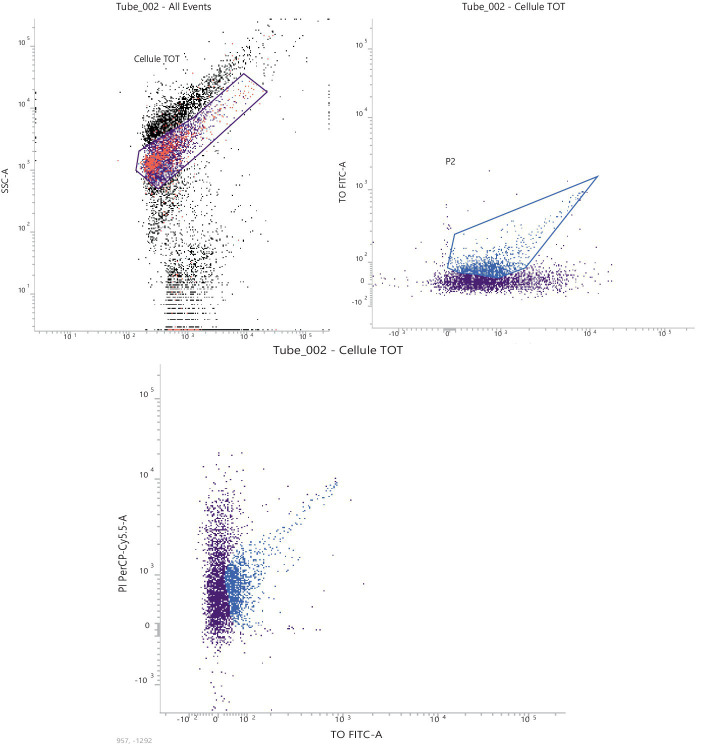
Representative graph of the 1,0E-07 dilution, corresponding to 2,46E + 11 FU/g.

The 1,0E-07 dilution, corresponding approx. to 1,0E+04 stained cells, was the most compliant dilution according to the microscopic count. The characteristic PI-TO dot plots obtained (see FSC-SSC gate) are shown in Figure 2. The 1,00E-07 dilution highlighted the cell scatter from the background of the matrix effect.

### 3.2 Enumeration of tyndallized probiotics by flow cytometry

To verify the applicability of this type of analysis in the field of food supplement quality control and releases, different batches of two raw materials and two finished products were acquired. At least three independent analyses of each product were performed.

Results obtained from raw material analysis, expressed as FU/g, are reported in Table 2.

**Table 2:** Results of cytometric analysis of raw materials.

Probiotic raw material	Batch	Replicate 1	Replicate 2	Replicate 3
Tyndallized *L. acidophilus*	A	1,27E+11	1,24E+11	1,25E+11
Tyndallized immuno blend	A	1,71E+11	1,78E+11	1,71E+11
	B	1,73E+11	1,76E+11	1,78E+11
	C	1,78E+11	1,79E+11	1,76E+11

Analytical data obtained from finished products, expressed as FU/mL, are reported in Table 3. The total count of each batch was compliant with the manufacturer’s specifications, confirming that the method we have optimized worked fine both on raw materials and on the finished product.

**Table 3:** Results of flow cytometric analysis of finished products containing tyndallized probiotics.

Finished product	Batch	Replicate 1	Replicate 2	Replicate 3
Food supplement, first formulation	A	2,87E+08	2,83E+08	2,78E+08
	B	2,69E+08	2,69E+08	2,59E+08
	C	2,81E+08	2,86E+08	2,83E+08
Food supplement, second formulation	A	2,74E+08	2,72E+08	2,77E+08
	B	2,86E+08	2,85E+08	2,80E+08
	C	2,81E+08	2,86E+08	2,86E+08
	D	2,73E+08	2,71E+08	2,71E+08

### 3.3 Method comparison

To compare the new method with the microscopy count declared in CoA, we used the ANOVA test both at the product-level and at the batch-level variations. Overall, the methods of analysis did not differ significantly. The counts obtained by the TO/PI were not significantly different from the *gold standard*.

Additionally, it was shown that the microscopic enumeration and the flow cytometric methods did not show statistically significant differences.

#### 3.3.1 Raw material

**Figure 3:**
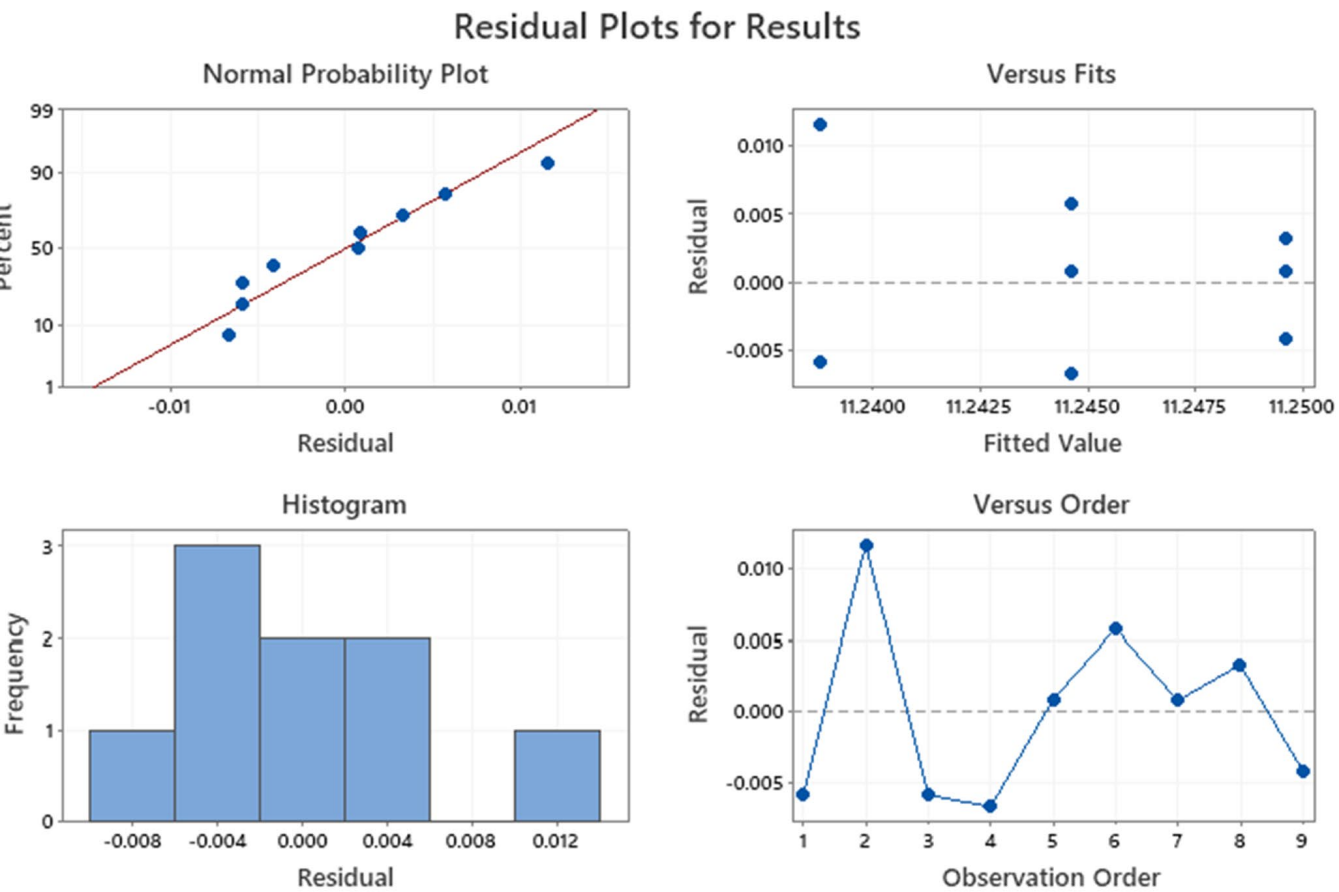
Residual diagnostic plots conducted on tyndallized immune blend formulations.

Before the comparison by ANOVA, we also verify the variance using Levene’s statistical test. The results indicated that variances were not significantly different. Then, a one-way ANOVA was applied to test raw data regarding the tyndallized immunoblend formulation (see Table 4). Briefly, ANOVA’s *p-value* was 0.259, confirming there were no significant differences between any pairs of measurements. The residuals diagnostic plots shown in Figure 3 demonstrated that the residuals are normally distributed and homoscedastic with respect to treatments, fitted values, and run order as required by the ANOVA method.

**Table 4:** Raw data of tyndallized immunoblend formulation.

Tyndallized immuno blend	*N*	Mean	StDev	95% CI
A	3	11.2388	0.0100	(11.2287; 11.2489)
B	3	11.2446	0.0062	(11.2345; 11.2548)
C	3	11.2496	0.0038	(11.2395; 11.2597)

#### 3.3.2 Finish products

The preliminary Levene’s test, conducted on raw data of Finished products, demonstrated that the variances of measures were not significantly different. Considering the p-value obtained from the ANOVA (*p* = 0.697; Table 5), we deduced there were no differences between the means of measurement.

**Table 5:** One-way ANOVA results.

Finished products	*N*	Mean	StDev	95% CI
Food supplement 1	9	8.44261	0.01508	(8.43406; 8.45116)
Food supplement 2	12	8.44475	0.00970	(8.43734; 8.45216)

**Figure 4:**
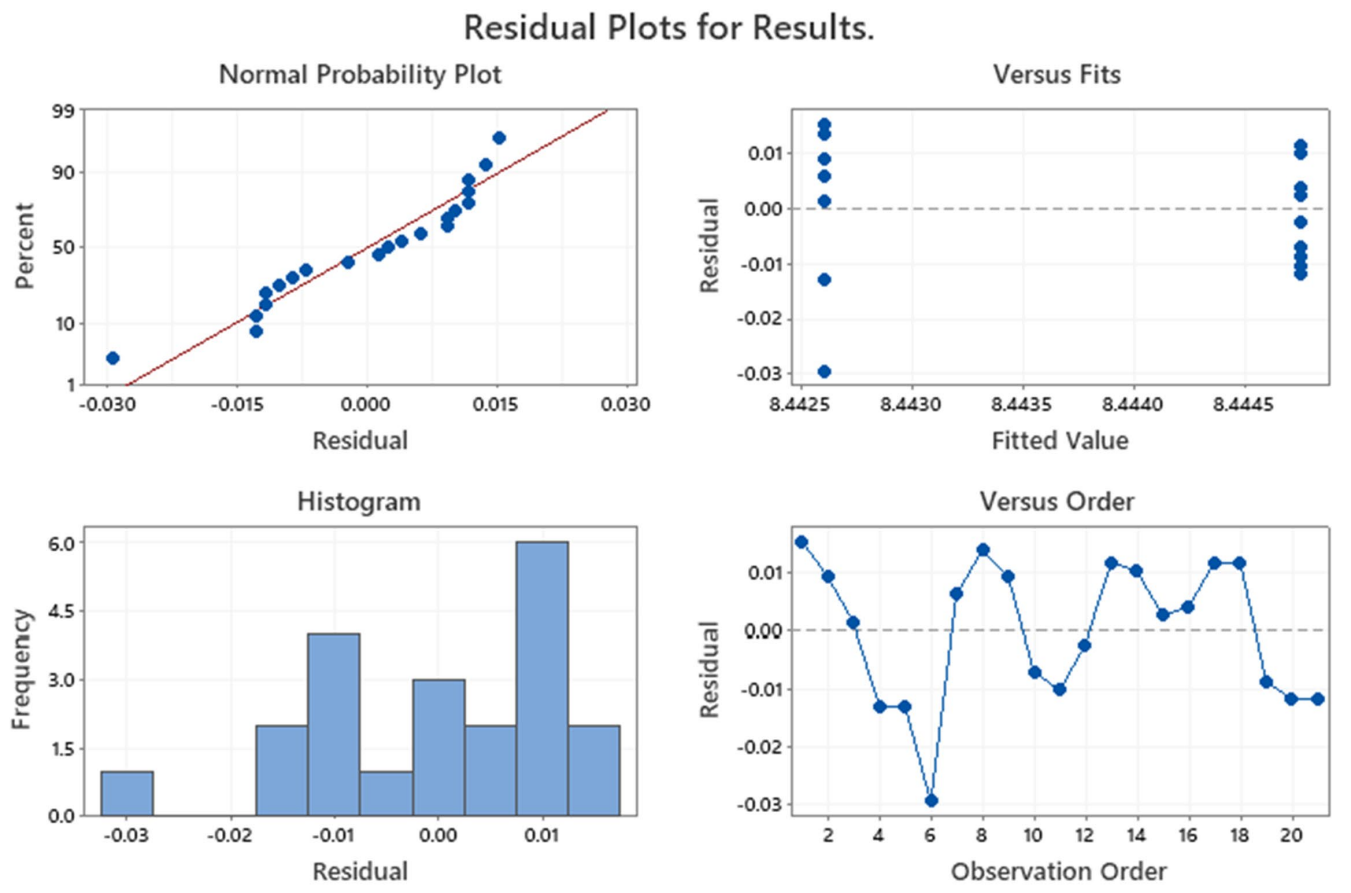
Residual diagnostic plots of tyndallized probiotics in Finished products.

The residual diagnostic plots of finished products are shown in Figure 4. Data indicated that the residuals were normally distributed and, in this case, homoscedastic with respect to treatments, fitted values, and run order, as required by the ANOVA method.

## 4 Discussion

The data obtained in this study represent the first consistent evidence of the analysis of *tyndallized probiotics* using the live/dead assay by flow cytometry.

Manufactured probiotic supplements are often analyzed using flow cytometry for a quality control assessment of the total viable cell in sample preparations, expressed as events/μL and converted in CFU/g (Amor et al., 2002; Díaz et al., 2010; Michelutti et al., 2020). The production of tyndallized bacteria by industries requires quality control assessment during all the manufacturing steps. For the determination of tyndallization efficacy and for dead-cell enumeration, no available methods are present (Bunthof and Abee, 2002; Davis, 2014; Marlicz et al., 2016; Ou et al., 2017; Burta et al., 2018; Jackson et al., 2019). Classical methods basically rely on the use of plate count methods, or microscopy counts, which are still regarded as the benchmark method for the determination of viability (Auty et al., 2001; Díaz et al., 2010; Davis, 2014; Hill et al., 2014; Jackson et al., 2019).

The definition of bacterial viability is associated with the intact cytoplasmic membrane, protein, and other cell components synthesis and energy production necessary to maintain an active metabolism, growth, and multiplication. Considering this definition, a variety of methodologies can provide a more comprehensive view of bacterial viability, bypassing the plate count method that selected only cultivable cells and not viable cells. Plate counting focuses solely on the growth and multiplication potential of a subset of the bacterial population without considering biochemical and metabolic processes.

Flow cytometry continues to become more conventional in the field of microbiology, independent of the bacterial species of interest. A limitation of ISO 19344 for the count and viability of probiotics with TO/PI staining is that it does not allow for differentiation between different species of probiotics.

In this study, we optimized a method for the quantification of dead probiotic strains in raw materials and finished products of food supplements.

This method is now used in our quality control laboratory and, to date, is the only available method for tyndallized bacterial count. We are one of the first laboratories to try to employ flow cytometry in the quality control assessment of dead probiotic products. This allows an easier, faster, and more accurate routine quality control method to count tyndallized products.

The value of this study is highlighted by the strong concordance between the microscopy count, the gold standard method, and our results.

Analyzing our data, we can see that the number of tyndallized bacteria is compliant with the CoA declared by the supplier. An ANOVA statistic was used to compare the number of dead bacteria counted, which allowed us to verify our new laboratory assay.

The method enumerations were compared both in raw materials and in finished products, considering their different compositions. In this way, the method showed its versatility.

We have shown that flow cytometry generates comparable results to microscopy counts. Regarding multi-strain products, enumeration by flow cytometry generated comparable counts to the gold standard method. The total number of tyndallized probiotic bacteria was likewise accurate, as declared in the CoA. Then, flow cytometry is an ideal method to assess this class of dead probiotics. A strain-differential count is an important requirement to determine and characterize benefits to the host that are specific for each strain. Since plate count often allows differentiation only between genera of bacteria and not between species (e.g., bifidobacteria), other techniques such as qPCR, digital PCR, and flow cytometry that can be useful in enumerating individual strains in a multi-strain blend using either plate count or flow cytometric methods remain a significant challenge.

Although the number of scientific reports reporting the application of flow cytometry for probiotic count is increasing, no relevant studies regarding tyndallized probiotics’ count are available (Amor et al., 2002; Assuncao et al., 2007; Díaz et al., 2010; Ashraf and Shah, 2011; Davis, 2014; Hill et al., 2014; Chiron et al., 2018; Jackson et al., 2019; Michelutti et al., 2020). Only a few studies have reported evidence of selective counting of probiotics by flow cytometry through the use of strain-specific antibodies, considering the complexity of monoclonal/polyclonal antibody production. In our laboratory, we focused on microbial cells’ physiological state using a double staining protocol, employing TO/PI for the evaluation of cellular metabolic activity. No commercial antibodies were available for testing species-specific staining, and we also referred to the morphological and viability status of raw materials and finished products.

SYTO-TO/PI staining is widely used to evaluate the physiological state of bacteria, while a three-color approach to SYTO-PI combined antibody is difficult to optimize. In fact, to reach this goal, it is necessary to create specific antibodies that recognize different species separately, and not all laboratories are equipped for this purpose. Furthermore, for quality control purposes, all reagents used for analysis must be certified and validated to guarantee robust and reproducible results.

Our approach facilitated quality control assessment of the microbial heterogeneity within food supplements at the single-cell level. We are currently working on improving this method for the release of all tyndallized probiotics produced in our industry.

## 5 Conclusion

Flow cytometry is a viable option for enumerating tyndallized cells for probiotic manufacturers.

The results obtained in this study indicate that flow cytometry is applicable for dead probiotic cell count assays, providing the analyst with additional information on the quality of the production. Bacterial counts obtained by flow cytometry were in agreement with the gold standard method, both in raw materials and in finished products.

This study improves time to result and standardization of count and provides new capabilities for in-line quality control testing to optimize production processes.

## Data availability statement

The raw data supporting the conclusions of this article will be made available by the authors, without undue reservation.

## Author contributions

VB: Data curation, Investigation, Methodology, Validation, Writing – original draft. MB: Methodology, Visualization, Writing – original draft, Writing – review & editing. MP: Formal analysis, Investigation, Methodology, Writing – original draft. AN: Data curation, Formal analysis, Investigation, Writing – review & editing. EN: Conceptualization, Data curation, Formal analysis, Funding acquisition, Investigation, Project administration, Supervision, Validation, Writing – review & editing.
